# Deterioration of duodenal lymphangiectasia after radiotherapy for gastric MALT lymphoma

**DOI:** 10.3332/ecancer.2017.752

**Published:** 2017-07-11

**Authors:** Masaya Iwamuro, Takehiro Tanaka, Hiromitsu Kanzaki, Seiji Kawano, Yoshiro Kawahara, Yoshiaki Iwasaki, Hiroyuki Okada

**Affiliations:** 1 Department of Gastroenterology and Hepatology, Okayama University Graduate School of Medicine, Dentistry, and Pharmaceutical Sciences, Okayama 700–8558, Japan; 2 Department of General Medicine, Okayama University Graduate School of Medicine, Dentistry, and Pharmaceutical Sciences, Okayama 700–8558, Japan; 3 Department of Pathology, Okayama University Hospital, Okayama 700–8558, Japan; 4 Department of Endoscopy, Okayama University Hospital, Okayama 700–8558, Japan; 5 Health Service Centre, Okayama University, Okayama 700–0082, Japan

**Keywords:** duodenal lymphangiectasia, radiotherapy, gastric neoplasms, gastrointestinal lymphoma

## Abstract

A 68-year-old Japanese woman underwent radiotherapy for gastric lymphoma. Although lymphangiectasia was sparsely observed in the second portion of the duodenum before radiotherapy, the number of pinpoint white spots obviously increased after the treatment. Although the duodenal lymphangiectasia gradually progressed, the patient had no features of protein-losing enteropathy. This case highlights the importance of endoscopic observation of the duodenum after irradiation to the abdomen as radiotherapy may secondarily cause intestinal lymphangiectasia.

## Introduction

Intestinal lymphangiectasia represents pathologic dilation of lymphatic vessels. Within the gastrointestinal tract, the most frequently reported site of lymphangiectasia is the duodenum [1, 2]. The majority of patients with duodenal lymphangiectasias are asymptomatic, but they may manifest malabsorption or protein-losing enteropathy in rare instances [3]. Macroscopically intestinal lymphangiectasias are most frequently observed as scattered pinpoint white spots [3]. It has been known that intestinal lymphangiectasia can secondarily emerge or progress in various settings, obstructing lymphatic flow. However, endoscopic images showing the process of emergence or progression have rarely been reported.

Here we present the endoscopic findings of a patient with duodenal lymphangiectasia. Oesophagogastroduodenoscopy revealed that the duodenal lesion progressively deteriorated after radiotherapy for the treatment of extranodal marginal zone lymphoma of the mucosa-associated lymphoid tissue (MALT lymphoma) in the stomach.

## Case presentation

**Figure 1.**
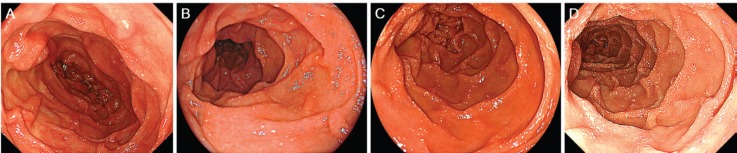
Oesophagogastroduodenoscopy images of the duodenum. Initially, white villi are sparsely observed in the second portion of the duodenum (A). Eight months later, the duodenal lymphangiectasia is unchanged (B). Endoscopy examinations performed six months (C) and twelve months (D) after radiotherapy show gradual progression of the lymphangiectasia.

**Figure 2.**
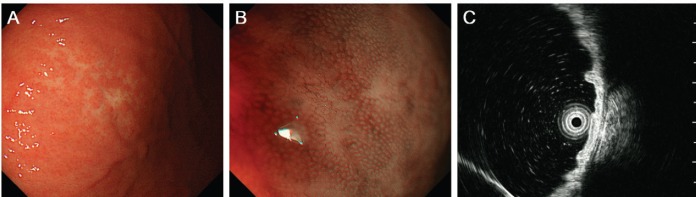
Oesophagogastroduodenoscopy images of the gastric MALT lymphoma. A pale lesion with indistinct boundaries is noted under white light observation (A) and magnifying observation with narrow-band imaging (B). Endoscopic ultrasonography confirms that the lymphoma is confined to the gastric mucosal layer (C).

A 68-year-old Japanese woman was admitted to our hospital to undergo radiotherapy for gastric MALT lymphoma. The patient had been attending our hospital as an outpatient for treatment of liver cirrhosis caused by the hepatitis C virus. She had periodically been undergoing oesophagogastroduodenoscopy to survey oesophageal varices. However, the varices had ruptured at 67 years of age and had been treated using endoscopic variceal ligation. Oesophagogastroduodenoscopy performed eight months before admission revealed sparse white villi in the second portion of the duodenum (Figure 1A). In addition, a pale lesion with indistinct boundaries was noted in the greater curvature of the gastric body (Figure 2A, B). A diagnosis of gastric MALT lymphoma was made based on the pathological analysis of the biopsy specimens from the gastric lesion. Endoscopic ultrasonography confirmed that the MALT lymphoma was confined to the gastric mucosal layer (Figure 2C). *Helicobacter pylori* was not detected in the stomach. Fluorescence *in situ* hybridisation analysis revealed no t(11;18)(q21;q21) API2/MALT1 translocation. Computed tomography imaging with contrast media and positron emission tomography showed no lymphoma involvement in other organs. Bone marrow biopsy showed no lymphoma cell infiltration. Consequently, the gastric MALT lymphoma was classified as stage I. Although pathological and serological examinations showed negative results for *H. pylori* infection, eradication therapy with amoxicillin, clarithromycin, and lansoprazole was performed. Since the gastric lesion remained unchanged after the eradication therapy, radiotherapy was planned for the treatment of MALT lymphoma.

**Figure 3.**
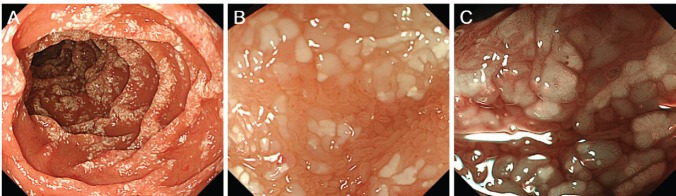
Oesophagogastroduodenoscopy images taken 55 months after radiotherapy show obviously worsened duodenal lymphangiectasia (A). Magnifying observation shows dilated, whitish duodenal villi (B). The margins of the villi are distinct. Magnifying observation with narrow-band imaging reveals elongated microvasculature within the villi (C).

**Figure 4.**
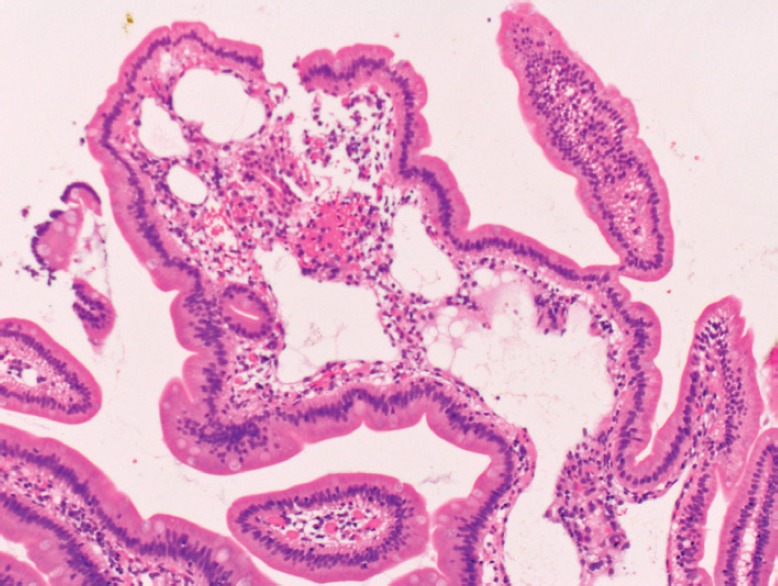
Pathological images of the duodenal lymphangiectasia. Biopsy examination reveals dilated lymphatic duct in the duodenal villi.

Physical examination revealed no lymphadenopathies. The liver and spleen of the patient were not palpable, and there was no edema on her legs. Laboratory examinations revealed pancytopenia (white blood cell count, 1630/mm^3^; hemoglobin, 10.8 g/dL, platelets 26,000/mm^3^). Her protein and albumin levels were within the normal ranges. Oesophagogastroduodenoscopy performed before radiotherapy revealed that the gastric MALT lymphoma and duodenal lymphangiectasia (Figure 1B) were unchanged since eight months. Radiotherapy was initiated, but the treatment was stopped after a total dose of 7.5 Gy as the pancytopenia worsened (white blood cell count, 860/mm^3^; platelets, 16,000/mm^3^). Biopsy sampling performed three months after radiotherapy revealed pathological remission of gastric MALT lymphoma. No recurrence was documented for the next 52 months. On the contrary, whitish villi in the duodenum increased in number with time (Figure 1C, D). Fifty-five months after radiotherapy, the duodenal lymphangiectasia obviously worsened (Figure 3). Biopsy revealed dilated lymphatic duct in the duodenal villi (Figure 4). The patient complained of no changes in her bowel habits.

## Discussion

Lymphangiectasia arises primarily or secondarily owing to obstruction of local lymphatic drainage. Acquired lymphangiectasis occurs as a result of various processes that cause scarring and lymphatic damage. In dermatology, cutaneous lymphangiectasia has been reported after treatment of breast, vulva, cervix, skin, and lung cancers [4, 5]. For instance, lymphangiectasia develops in the chest skin as clusters of translucent vesicles several years after mastectomy and radiation treatment for breast cancers [5].

Lymphangiectasia can occur in the gastrointestinal tract, particularly in the duodenum [2]. Although it may develop in relation to protein-losing enteropathy, the majority of cases of intestinal lymphangiectasia are believed to be asymptomatic and are incidentally identified during endoscopy examinations. Kim *et al* reported that in their retrospective review of 1866 patients who underwent oesophagogastroduodenoscopy, duodenal lymphangiectasia was endoscopically identified in 59 patients (3.2%) and histologically diagnosed in 35 patients (1.9%) [6]. None of the patients had clinical evidence of malabsorption or protein-losing enteropathy. Macroscopically, the duodenal lesions presented as scattered pinpoint white spots (40.0%), diffuse prominent whitish villi (31.4%), or focal small whitish macules or nodules (28.6%). Generally using magnifying observation with narrow-band imaging, enlarged villi and elongated microvessels can be observed in the white spots [7]. White spots of the lymphangiectasias are of homogenous sizes with clear margins. In the present patient, although scattered pinpoint white spots were sparsely observed in the second portion of the duodenum before radiotherapy, endoscopic features of lymphangiectasia were more evident after the treatment of gastric MALT lymphoma.

Nakano *et al* reported a postmortem case that presented with protein-losing enteropathy because of intestinal lymphangiectasia [8]. The patient had developed hypoproteinemia five years after a radical operation and radiotherapy for oesophageal carcinoma. Autopsy examination revealed duodenal and small intestinal lymphatic dilatation and fibrosis of the mesenteric lymph node. Therefore, it was speculated that surgery and radiotherapy led to mesenteric lymph node fibrosis and the subsequent lymphatic congestion. Rao *et al* also described a patient who developed intestinal lymphangiectasia after radiotherapy and chemotherapy for metastatic testicular teratoma [9]. Other cases wherein patients presented with malabsorption after radiotherapy have been reported by several authors [10, 11]. These cases indicate that intestinal lymphangiectasia may develop after surgical and/or radiological damage as cutaneous lesions. However, as endoscopy examinations were not performed in the previously reported cases to the best of our knowledge, the present case report is the first to describe macroscopic images of duodenal lymphangiectasia that developed after radiotherapy.

Pathologies causing secondary intestinal lymphangiectasia include sarcoidosis [12], tuberculosis [13], eosinophilic gastroenteritis, systemic lupus erythematosus, systemic sclerosis [14], retroperitoneal fibrosis, and constrictive pericarditis [9, 15]. In addition, lymphomas reportedly cause secondary intestinal lymphangiectasia as a result of anatomical or dynamic alterations of the lymphatic flow [16, 17]. Although the present patient had gastric MALT lymphoma, it was unlikely that the lymphoma was the main etiological factor of the duodenal lymphangiectasia because lymphangiectasia progressed after achieving complete remission of lymphoma by irradiation. In previously reported cases protein-losing enteropathy with intestinal lymphangiectasia were resolved after treatment of lymphoma [2, 18–20] which is inconsistent with the result observed in the present patient.

## Conclusions

We observed a case of duodenal lymphangiectasia secondary to radiotherapy. Oesophagogastroduodenoscopy showed that the lymphangiectasia gradually progressed as time passed. Although the present patient was asymptomatic, this case underscores the importance of endoscopic observation of the duodenum after irradiation to the abdomen because such treatment may cause intestinal lymphangiectasia and subsequent protein-losing enteropathy.

## Abbreviations

MALT lymphoma: extranodal marginal zone lymphoma of the mucosa-associated lymphoid tissue

## Conflicts of interest

The authors have no conflicts of interest to declare.
